# Arthroscopic Distal Clavicle Bone Bock Combined With Hill-Sachs Remplissage for Primary Anterior Shoulder Instability Treatment

**DOI:** 10.1016/j.eats.2023.11.010

**Published:** 2024-01-01

**Authors:** Nicolas Bonnevialle, Antoine Baltassat, Vincent Martinel, Hugo Barret, Pierre Mansat

**Affiliations:** a Service de Chirurgie Orthopédique et Traumatologique, CHU de Toulouse, France; b Clinique Universitaire du Sport, Toulouse, France; c Institut de Recherche Riquet (I2R), Toulouse, France; d Clinique Pyrénées-Ormeau, Groupe Elsan, Tarbes, France

## Abstract

Arthroscopic distal clavicle transfer is an effective option to treat anterior shoulder instability with glenoid bone loss. The use of this free bone graft in an all-inside procedure, with a cortical button fixation makes the construct simpler to perform and allows to be combined with a Hill-Sachs Remplissage to address humeral bone defect. The morbidity of the donor site is low and provide the biologic capacity of an autograft. We report a step-by-step procedure, and the rationale are discussed.

Anterior shoulder instability associated with bipolar bone lesion may be treated with anterior glenoid bone block and Hill-Sachs Remplissage. If arthroscopic Latarjet procedure is evolving for a long time, it remains a demanding technique that cannot be offered to all shoulder-trained surgeons.^1^, ^2^, ^3^, ^4^, ^5^ On the other hand, free bone grafts may be an alternative, with a superiority for autograft in term of safety and biologic patterns over allograft.^6^

Since 2014, Tokish et al.^7^ popularized the use of distal clavicle in this indication arguing a low morbidity of the donor site, as well as the availability for both bone and chondral reconstruction. In this line, Boileau et al.^8^ described a full-arthroscopic technique indicated in cases of revision of a failed Latarjet procedure with a satisfactory correction of glenoid bone loss using the concave side of the lateral clavicle, fixed in the “standing” position with a cortical button.

This Technical Note aimed to report a procedure using a similar guided system in a primary case, in association with a Hill-Sachs Remplissage to address a bipolar bone loss.

## Ethical Approval

This Technical Note was approved by our ethical committee, and a written informed consent was obtained from the patient filmed (RnIPH 2023-032).

## Surgical Technique

Video 1 demonstrates the surgical technique. The goal of the procedure is to address arthroscopically:

- glenoid bone loss with an autograft harvested from lateral clavicle, transferred, and fixed onto the glenoid neck with a cortical button
- humeral bone loss with Hill-Sachs Remplissage procedure

## Patient Positioning and Surgical Field Preparation

The patient is placed in the beach chair position, with the trunk flexed at 30° to 45°, under general anaesthesia and interscalene block. Antibioprophylaxy of 2 g of intravenous (IV) Cephalosporin is injected 30 min before skin incision, as well as 1 g of IV acid tranexamic to decrease intraoperative bleeding. The head of the patient is fixed with a head rest and the arm is secured with a mobile harm holder (Trimano Fortis Support Arm, Maquet [Arthrex]), without any traction. The surgical field is prepared with standard fashion and is draped above the sternum in front and to the medial border of the scapula in the back (Custom pack shoulder, Mölnlycke).

**Fig 1:**
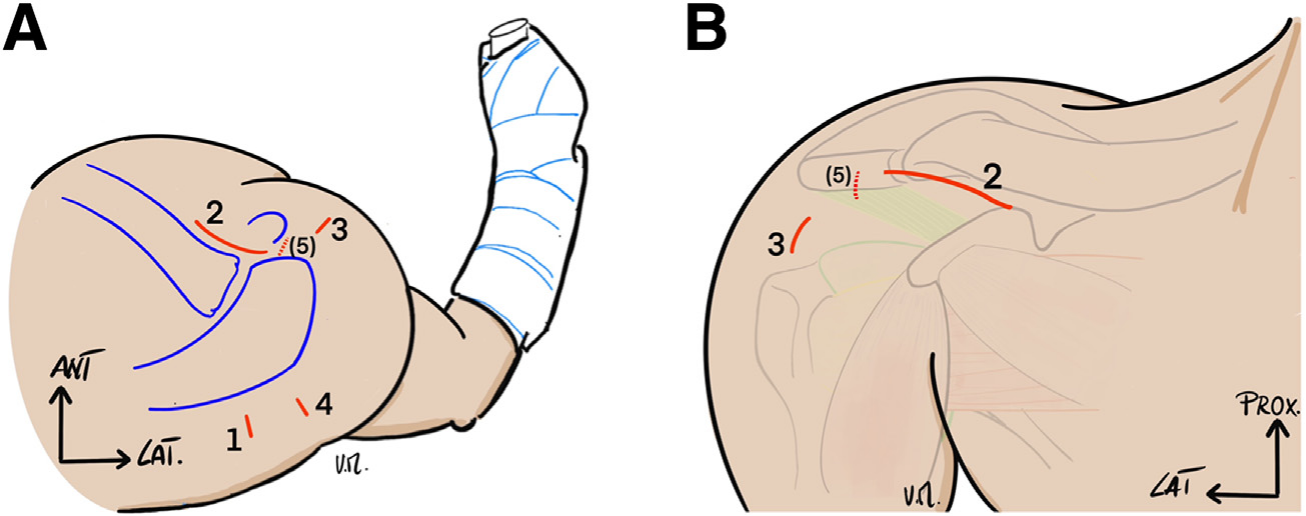
(A and B) Arthroscopic portals and mini-open approach of a patient in the beach chair position (right shoulder). 1 denotes the posterior soft point viewing portal; 2 denotes the mini-open clavicular approach for clavicular harvesting; it is placed 1 cm anteriorly to the acromioclavicular joint on 2 at 3 cm long. 3 denotes anterior-lateral instrumental portal placed at mid-distance between the anterior-lateral angle of the acromion and the axillary fold; 4 denotes posterior-lateral portal for Hill-Sachs Remplissage placed at around 2 cm inferior to the posterior angle of the acromion (outside-in technique); 5 denotes accessory anterior-medial portal for rotator interval release.

The bony landmarks of the shoulder are drawn; the approach for clavicle harvesting is placed 1 cm anteriorly to the acromioclavicular joint at 2 to 3 cm long. A standard posterior arthroscopic portal is identified in the soft-point, and one more anterior-lateral portal is placed a mid-distance between the anterior-lateral angle of the acromion and the axillary fold. An accessory anterior-medial portal may be necessary, depending on the patient’s muscular shape body (Fig 1).

### Step 1: Graft Harvesting and Preparation

A skin incision is performed anteriorly to lateral clavicle, as previously described, for mini-open approach. The acromioclavicular joint is identified with a needle, and the trapeziodeltoid fascia is detached medially from the clavicle to visualize its last 2 cm. A standard bended saw helps to cut at 1 cm the lateral clavicle perpendicular to main axis. The graft is then detached properly from the soft tissue and the inferior concave part colored with a skin pen. It is sent to the back table, where a glenoid guide is used to place the cortical button (Latarjet Guiding System, Smith & Nephew, Andover, MA). The specific 2.8-mm cannulated drill bit with outer sleeve aimed to shuttle the preloaded device (Osteo-connect; Smith & Nephew, Andover, MA). The peg-button is placed on the articular surface to fix the graft onto the glenoid rim by the cut side later on. The graft is remodeled with a rongeur on demand to allow it to freely slide into a rigid canula with a 15-mm external diameter.

### Step 2: Opening Rotator Interval and Glenoid Preparation

A 70° scope is introduced from the posterior portal, and a diagnostic arthroscopy is performed. The mini-open anterior clavicle approach is used to introduce the cautery (EDGE, Conmed) and release fully the rotator interval. As mentioned above, an accessory anterior-medial portal may be necessary to facilitate the access to the rotator interval, depending on the deltoid belly obstruction. A skin clamp is used to close partially the mini-open approach and avoid a leak of fluid. The remnant labrum is then mobilized from the glenoid rim, from 1 to 6 o’clock position. Pay attention is required to preserve the anterior capsule attached to the labrum. The instrumental anterior-lateral portal is useful at this step to introduce a stick and push away the capsular-labral complex. A PDS suture is passed through the capsule at 4 to 5 o’clock position to shift the capsule at the end of the procedure and facilitate the transfer of the graft. The glenoid neck is then flattened and abraded with a motorized rasp (Smith & Nephew, Andover, MA).

### Step 3: Glenoid Drilling

The scope is switched from the posterior to the anterior working portal. A posterior switching-stick helps to slide a half-pipe along with the dedicated glenoid guide can be positioned inside the joint. The hook is placed at 4 o’clock, and the guide must be flush to the glenoid surface. When it is secured against the posterior glenoid surface, the 2.8-mm cannulated drill perforates the glenoid from posterior to anterior under arthroscopic control. The outer sleeve is maintained in place when the drill is removed, allowing to push a PDS suture inside.

### Step 4: Graft Transfer and Fixation

**Fig 2:**
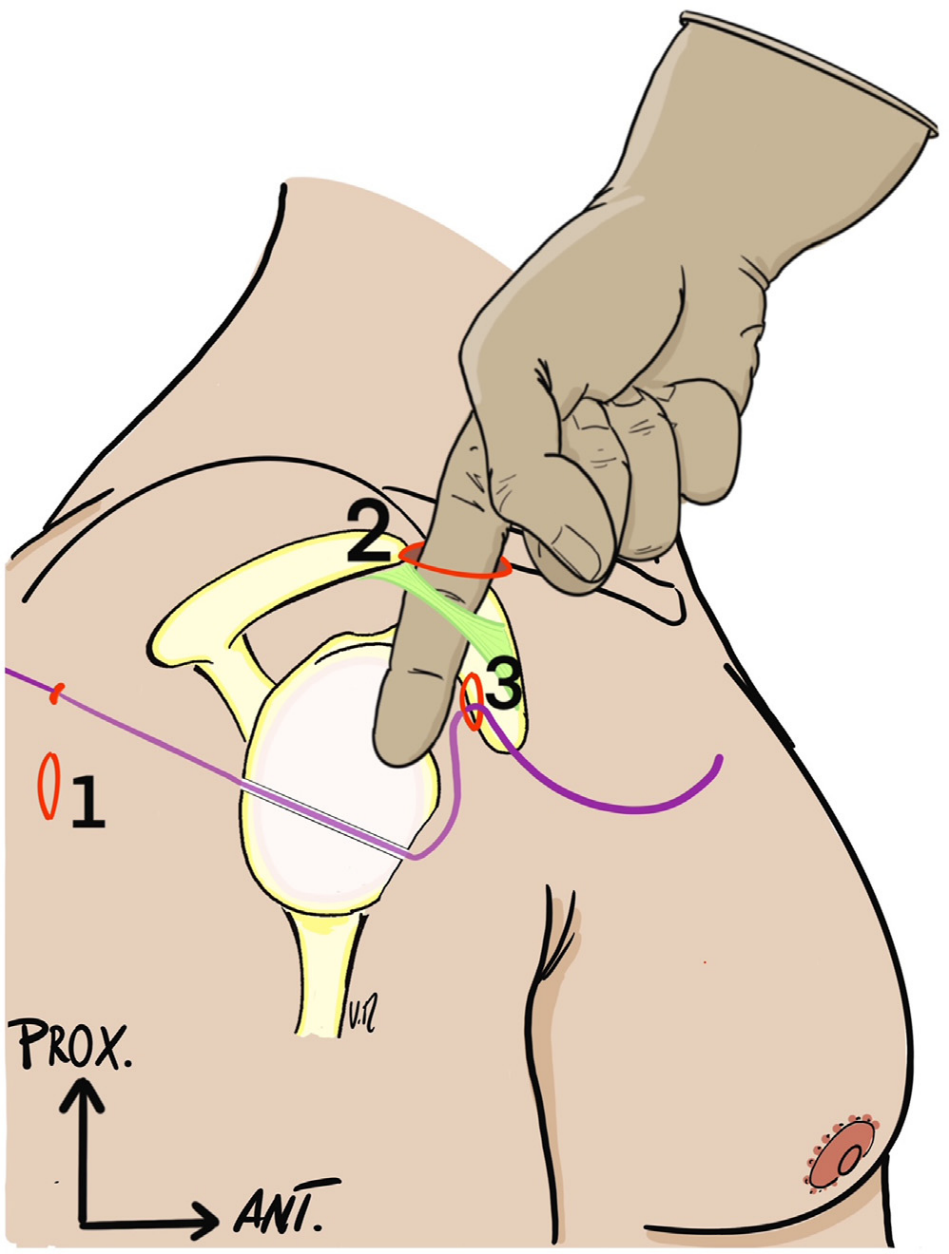
Finger dissection through the mini-open clavicular approach to prepare the graft transfer via a canula (right shoulder in a beach chair position). The finger is pushed firmly in the rotator interval previously opened and must reach the gleno-humeral joint under the control of the scope placed in posterior viewing portal.

**Fig 3:**
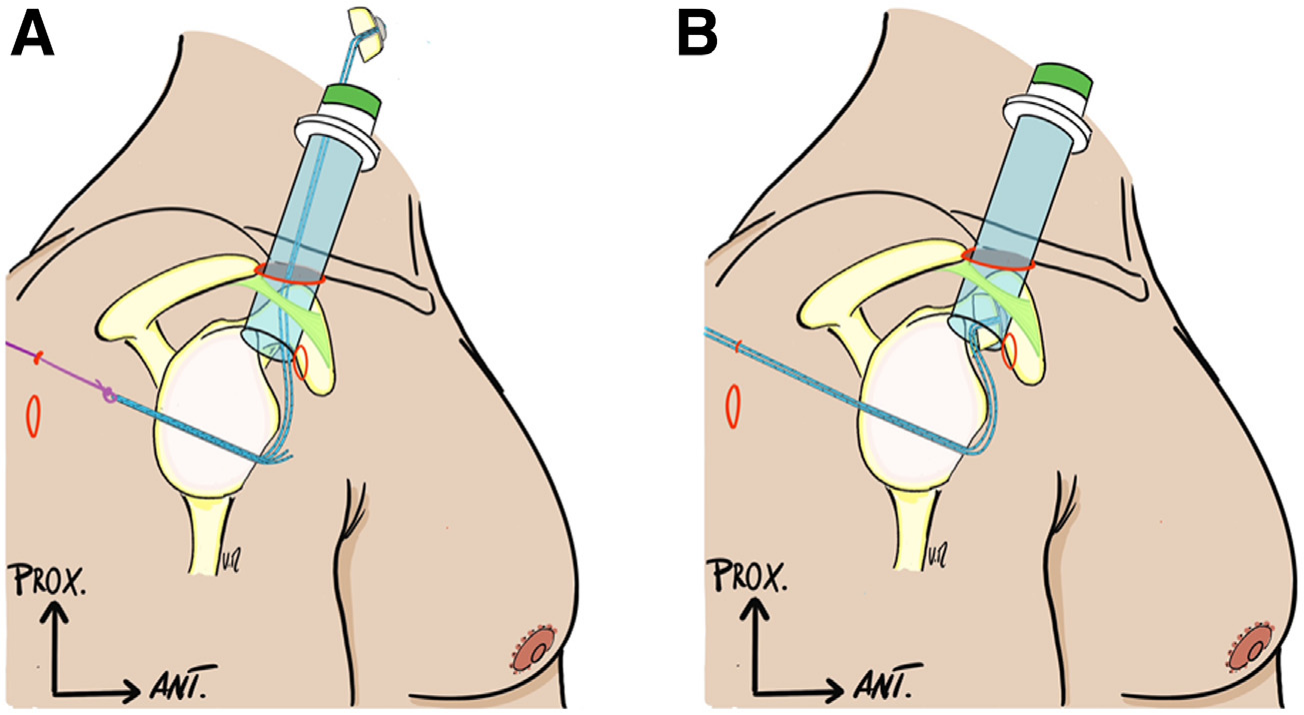
(A and B) A 15-mm diameter canula is placed in the track of the finger (right shoulder in a beach chair position). A grasper retrieves the PDS of the glenoid outer sleeve and shuttles the tail ends of the cortical button through the canula. Bone block is transferred gently, by pulling on the tail ends under the control of the scope in the posterior viewing portal.

The scope is then switched back from anterior to posterior. To place the 15-mm canula, the “finger dissection” is performed using the mini-open clavicle approach until fingertip visualization (Fig 2). The canula is easily introduced along the same slope into the joint, through the rotator interval. A grasper retrieves the PDS of the outer sleeve and shuttles the tail ends of the cortical button through the canula. The outer sleeve is removed, the suture tapes of the cortical button are pulled, and the graft slides gently onto the glenoid neck (Fig 3). A hook or grasper is useful to place the free bone graft in the correct position, the colored curved cortex is in the line of the articular glenoid surface and parallel to the glenoid rim. A posterior button is slid along the posterior free loop suture, and a Nice-knot is performed. A suture tensioner is applied 3 times at 100 N, to optimize graft compression before locking the Nice-knot with 3 square knots.

### Step 5: Bankart repair + Hill-Sachs Remplissage

1 first knotless 1.8-mm all-suture knotless anchor (Fibertak, Arthrex) is placed through the anterior-lateral portal bellow the bone graft at the 5 o’ clock position. A suture passer helps to load the capsular-labral complex, and this first anchorage is left ready to be tightened at the end of this step.

**Fig 4:**
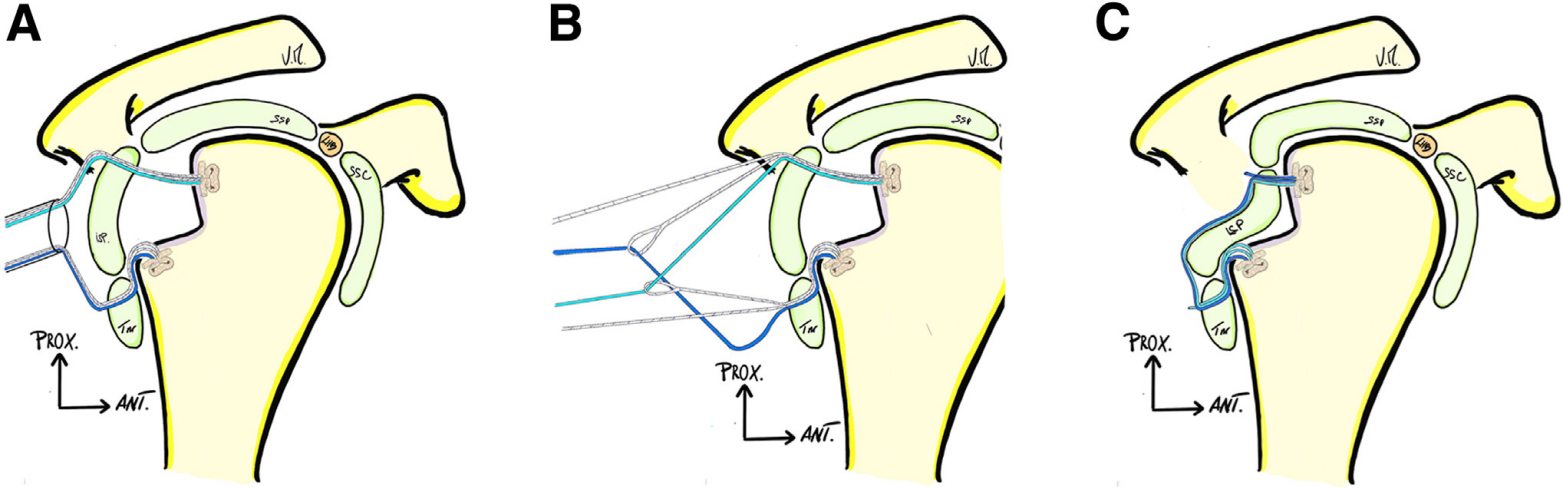
Hill-Sachs Remplissage step (right shoulder). (A) Through the posterior-lateral working portal, 2 knotless all-suture FiberTak (Arthrex) 1.8-mm anchors are placed into the humeral bone defect. (B) Interconnection of the 2 anchors is performed allowing a bridging reconstruction. (C) Final aspect after the 2 anchors have been locked.

Thanks to the 70° scope, the engaging Hill-Sachs lesion is visualized, and a posterior lateral portal is made at the appropriate position with the help of spinal needle. A posterior 5.5-mm cannula (Clear-Trac Flexible, Smith & Nephew) is pushed under the deltoid, but outside the joint. The anchor-guide is introduced inside the joint, at the lower part of the humeral defect, refreshes the Hill-Sachs lesion, and allows the surgeon to place the first knotless, 1.8-mm all-suture knotless anchor (Fibertak, Arthrex). In the same manner, a second knotless 1.8-mm all-suture knotless anchor is placed at the top of the Hill-Sachs lesion. The 2 anchors are connected to each other by crossing the sutures, aiming to perform a capsule-tenodesis-bridged Remplissage (Fig 4, A and B).

**Fig 5:**
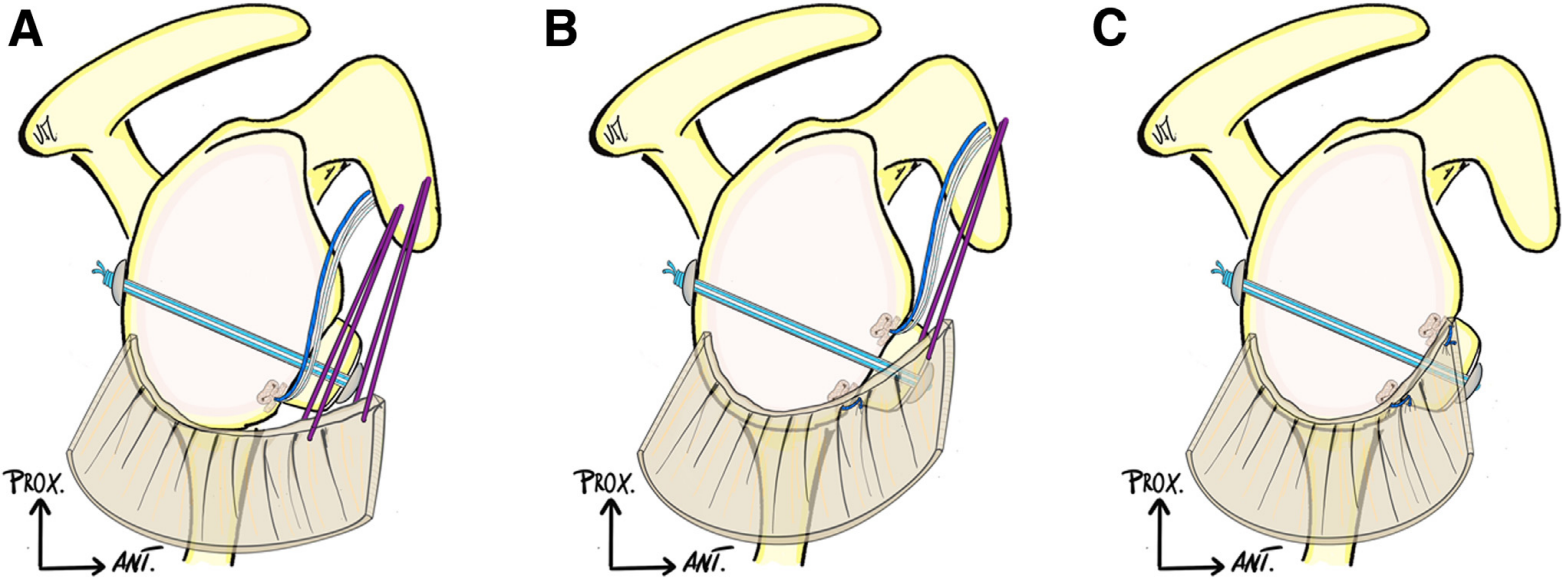
Bankart repair and capsular shift step (right shoulder). (A) 1st inferior, all-suture knotless anchor placement below the graft with 2 PDS suture ready to shuttle. (B) First anchor is locked, and the second is placed above the graft. (C) Final construct.

A second (and possible third) knotless 1.8-mm, all-suture knotless anchor is impacted above the graft at 3 to 2 o’clock and the previous PDS suture (placed at step 2) is used to shift the capsule from south to north to conclude the Bankart repair (Fig 5).

Finally, the 2 anchors of the remplissage are locked and the capsulotenodesis achieved (Fig 4C).

### Postoperative Management

The approaches are closed with absorbable intradermal overlock. The arm is placed in a sling for 3 weeks. Pendulum exercises are allowed immediately, as well as active range of motion recovery in anterior elevation. External rotation is protected to 1.5 months. Sports at risk for the shoulder are allowed as soon as the graft is healed, full range of motion is recovered, and muscular strength is found satisfactory.

## Discussion

Anterior shoulder instability may be associated with bipolar bone lesions. In cases of significant glenoid bone loss, Latarjet procedure is a current option to address bone loss, aiming to reconstruct the anatomy and adding a sling effect to stabilize the shoulder.^3^ Arthroscopic Latarjet has been successfully introduced for more than 10 years but exposes to a long learning curve, limiting the popularity to surgeons with a high volume of patients and widely comfortable with arthroscopic techniques.^1^^,^^2^^,^^4^^,^^5^ In another hand, arthroscopic tool helps to address all of the lesion (cuff tears, loose bodies, Bankart lesion, SLAP lesion, and humeral bone loss) that can be related to anterior shoulder instability and lower postoperative rate of short-term complications.^9^

Free bone graft has been proved to provide equivalent clinical outcomes to Latarjet.^10^ Despite an optimized biologic capacity, the iliac crest bone graft is encumbered of painful donor site with a worrisome rate of morbidity.^11^^,^^12^ More recently, the lateral part of the clavicle was proposed as an alternative to the iliac crest, providing a geometry that allows to reconstruct more than 20% of the glenoid surface.^7^^,^^8^^,^^13^ It was advocated that because of the articular surface of the distal clavicle, it could be possible to associate bone and chondral reconstruction.^7^ However, the fixation of this graft, as well as the positioning remained a technical issue under arthroscopy.^14^ The use of the cortical button-guided option has been proposed as an alternative of classic screw fixation, avoiding any impingement with the surrounding tissue or neurologic injury, and a reproducible placement of the device.^2^^,^^4^ Biomechanically, the stability of cortical button is exposed to criticism in case of Latarjet procedure.^15^ However, a free bone graft does not act in the same manner because no pullout loads are applied through the conjoint tendon, especially in external rotation.^16^^,^^17^ Moreover, cyclic loading of the cortical button with a suture tensioner avoids creep of the sutures and late elongation.^17^ It aims also to optimize compression, without any risk of bone block breakage.^18^^,^^19^ Pearls and pitfalls of the surgical technique are summarized in Table 1.

**Table 1:** Pearls and Pitfalls

Pearls	Pitfalls
1. Anterior approach of the lateral clavicle to reuse it for graft transfer 2. Large opening of the rotator interval, anterior capsular-labral complex release, and “finger dissection” allows the surgeon to place the cannula easily. 3. Proper flattening of the glenoid surface matches the cut side of the graft. 4. Use the guided system for glenoid and graft drilling to get the same offset of the cortical button fixation. 5. Cyclic loading of the cortical button with a suture tensioner avoids creep of the sutures.	1. A too-big lateral clavicle harvest (>1 cm) risks making the transfer tough. 2. Posttraumatic osteolysis of the lateral clavicle may be an issue and must be evaluated preoperatively.

In addition to glenoid bone grafting, this arthroscopic technique allows to address humeral bone loss with a Hill-Sachs Remplissage and anterior soft tissue lesion with a Bankart repair. It has been reported that combining the procedures enhance the stability of the shoulder, especially in case of off-track lesion.^20^ Using a double-pulley technique with interconnected anchors makes the Remplissage simpler and more reproducible, without any compromise of biomechanical patterns.^21^^,^^22^ Finally, the Bankart repair makes the graft extra-articular and tightens anterior inferior capsule in the same time. Advantages and disadvantages of the surgical technique are reported in Table 2.

**Table 2:** Advantages and Disadvantages of the Technique

Advantages	Disadvantages
1. No donor site morbidity (provided the harvest is 1 cm) 2. All-inside technique to address glenoid and humeral bone loss 3. Short learning curve and accessibility to standard fellow-trained surgeons 4. Graft preparation on the back table (by an assistant), while the procedure is going forward by the main surgeon 5. Guided technique for graft positioning 6. Cortical button avoiding hardware impingement requiring potentially to be removed	1. Biomechanical limited effect without the sling effect of a coracoid transfer met with Latarjet construct 2. Limited graft size 3. Sacrifice of a normal structure 4. Bone density variability of the lateral clavicle

To conclude, our technique fulfilled all specifications of an arthroscopic procedure, combining a bone block transfer to address glenoid bone loss, a Hill-Sachs remplissage to address humeral bone loss, with a low morbidity of the graft harvest site. This all-inside procedure should be more reproducible to shoulder-trained surgeons, without requiring a long learning curve, but need the proof of long-term clinical evaluation.

## Disclosures

The authors report no conflicts of interest in the authorship and publication of this article. Full ICMJE author disclosure forms are available for this article online, as supplementary material.

## Supplementary Data

ICMJE author disclosure forms

Video 1 Surgical technique of lateral clavicle bone block + Hill-Sachs Remplissage.
