# Erratum

**DOI:** 10.1016/j.eats.2024.103212

**Published:** 2024-09-17

In the article, “Arthroscopic Distal Clavicle Bone Bock Combined With Hill-Sachs Remplissage for Primary Anterior Shoulder Instability Treatment” published in the March 2024 issue (*Arthroscopy Techniques* 2024;13:102882), the word “Block” in the title was misspelled. The article has since been corrected online.

