# Supplementary material for: Arthroscopic Distal Clavicle Bone Bock Combined With Hill-Sachs Remplissage for Primary Anterior Shoulder Instability Treatment
Source: Arthrosc Tech. 2024 Jan 1;13(3):102882. doi: 10.1016/j.eats.2023.11.010 (PMC10995697; doi:10.1016/j.eats.2023.11.010)
Supplement: ICMJE author disclosure forms [file mmc1.pdf]

Arthroscopy: The Journal of Arthroscopic and Related Surgery

— Instructions —

ICMJE Form for Disclosure of Potential Conflicts of Interest

Each author of the manuscript must separately complete and save this form using his or her name in the file name. Each author's completed form must then be uploaded with the manuscript.

The purpose of this form is to provide readers of your manuscript with information about your other interests that could influence how they receive and understand your work. The form is in four parts:

Section 1. Identifying information

Enter your full name and provide the manuscript title.

Section 2. The work under consideration for publication

This section asks for information about the work that you have submitted for publication. The time frame for this reporting is that of the work itself, from the initial conception and planning to the present. The requested information is about resources that you received, either directly or indirectly (via your institution), to enable you to complete the work. Checking "No" means that you did the work without receiving any financial support from any third party — that is, the work was supported by funds from the same institution that pays your salary and that institution did not receive third-party funds with which to pay you. If you or your institution received funds from a third party to support the work, such as a government granting agency, charitable foundation, or commercial sponsor, check "Yes." Then complete the provide the information requested.

Section 3. Relevant financial activities outside the submitted work

This section asks about your financial relationships with entities in the biomedical arena that could be perceived to influence, or that give the appearance of potentially influencing, what you wrote in the submitted work. You should disclose interactions with ANY entity that could be considered broadly relevant to the work.

Report all sources of revenue paid (or promised to be paid) directly to you or your institution on your behalf over the 36 months prior to submission of the work. This should include all monies from sources with relevance to the submitted work, not just monies from the entity that sponsored the research. Please note that your interactions with the work's sponsor that are outside the submitted work should also be listed here. If there is any question, it is usually better to disclose a relationship than not to do so.

For grants you have received for work outside the submitted work, you should disclose support ONLY from entities that could be perceived to be affected financially by the published work, such as entities or foundations supported by entities that could be perceived to have a financial stake in the outcome. Public funding sources, such as government agencies, charitable foundations, or academic institutions, need not be disclosed. For example, if a government agency sponsored a study in which you have been involved and drugs were provided by a pharmaceutical company, you need only list the pharmaceutical company.

Section 4. Other relationships

Use this section to report other relationships or activities that readers could perceive to have influenced, or that give the appearance of potentially influencing, what you wrote in the submitted work.

ICMJE Form for Disclosure of Potential Conflicts of Interest

Section 1. Identifying Information

1. Given Name BARRET ________
2. Surname HUGO__________________
3. Are you the corresponding author? NO
4. Effective Date 19/08/2023___________________________
5. Manuscript Title: **Arthroscopic distal clavicle bone block combined with Hill-Sachs remplissage for primary anterior shoulder instability treatment**

Section 2. The Work Under Consideration for Publication

Did you or your institution at any time receive payment or services from a third party for any aspect of the submitted work (including but not limited to grants, data monitoring board, study design, manuscript preparation, statistical analysis, etc…)?

Complete each item by typing an X in answer yes or not and completing the information requested if an answer is Yes. If you have more than one relationship, add lines.

1. Grant

___No ___ _

2. Consulting fee or honorarium

___No

3. Support for travel to meetings for the study or other purposes

___No

4. Fees for participation in review activities such as data monitoring boards, statistical analysis, end-point committees, and the like

___No

5. Payment for writing or reviewing the manuscript

___No

6. Provision of writing assistance, medicines, equipment, or administrative support

___No

7. Other

___No

* This means money that your institution received for your efforts on this study.
† Use this section to provide any needed explanation.

Section 3. Relevant financial activities outside the submitted work

1. Board membership

___No

2. Consultancy

No

3. Employment

___No

4. Expert testimony

___No

5. Grants/grants pending

___No

6. Payment for lectures including service on speakers bureaus

___No

7. Payment for manuscript preparation

___No

8. Patents (planned, pending or issued)

___No

9. Royalties

___No

10. Payment for development of educational presentations

___No

11. Stock/stock options

___No __

12. Travel/accommodations/ meeting expenses unrelated to activities listed**

___No

13. Other (err on the side of full disclosure)

___No

** For example, if you report a consultancy above there is no need to report travel related to that consultancy on this line.

Section 4. Other relationships

Are there other relationships or activities that readers could perceive to have influenced, or that give the appearance of potentially influencing, what you wrote in the submitted work?

___No other relationships/conditions/circumstances that present a potential conflict of interest

>>>>>>>>>>><<<<<<<<<<<

The International Committee of Medical Journal Editors

The ICMJE Disclosure of Potential Conflicts of Interest Form was adopted by *Arthroscopy: The Journal of Arthroscopic and Related Surgery* along with 17 other leading orthopaedic journals at the 2011 annual meeting of the American Academy of Orthopaedic Surgeons.

Arthroscopy: The Journal of Arthroscopic and Related Surgery

— Instructions —

ICMJE Form for Disclosure of Potential Conflicts of Interest

Each author of the manuscript must separately complete and save this form using his or her name in the file name. Each author's completed form must then be uploaded with the manuscript.

The purpose of this form is to provide readers of your manuscript with information about your other interests that could influence how they receive and understand your work. The form is in four parts:

Section 1. Identifying information

Enter your full name and provide the manuscript title.

Section 2. The work under consideration for publication

This section asks for information about the work that you have submitted for publication. The time frame for this reporting is that of the work itself, from the initial conception and planning to the present. The requested information is about resources that you received, either directly or indirectly (via your institution), to enable you to complete the work. Checking "No" means that you did the work without receiving any financial support from any third party — that is, the work was supported by funds from the

same institution that pays your salary and that institution did not receive third-party funds with which to pay you. If you or your institution received funds from a third party to support the work, such as a government granting agency, charitable foundation, or commercial sponsor, check "Yes." Then complete the provide the information requested.

Section 3. Relevant financial activities outside the submitted work

This section asks about your financial relationships with entities in the biomedical arena that could be perceived to influence, or that give the appearance of potentially influencing, what you wrote in the submitted work. You should disclose interactions with ANY entity that could be considered broadly relevant to the work.

Report all sources of revenue paid (or promised to be paid) directly to you or your institution on your behalf over the 36 months prior to submission of the work. This should include all monies from sources with relevance to the submitted work, not just monies from the entity that sponsored the research. Please note that your interactions with the work's sponsor that are outside the submitted work should also be listed here. If there is any question, it is usually better to disclose a relationship than not to do so.

For grants you have received for work outside the submitted work, you should disclose support ONLY from entities that could be perceived to be affected financially by the published work, such as entities or foundations supported by entities that could be perceived to have a financial stake in the outcome. Public funding sources, such as government agencies, charitable foundations, or academic institutions, need not be disclosed. For example, if a government agency sponsored a study in which you have been involved and drugs were provided by a pharmaceutical company, you need only list the pharmaceutical company.

Section 4. Other relationships

Use this section to report other relationships or activities that readers could perceive to have influenced, or that give the appearance of potentially influencing, what you wrote in the submitted work.

ICMJE Form for Disclosure of Potential Conflicts of Interest

Section 1. Identifying Information

1. Given Name BONNEVIALLE ________
2. Surname NICOLAS___________________
3. Are you the corresponding author? Yes
4. Effective Date 19/08/2023____________________________
5. Manuscript Title: **Arthroscopic distal clavicle bone block combined with Hill-Sachs remplissage for primary anterior shoulder instability treatment**

Section 2. The Work Under Consideration for Publication

Did you or your institution at any time receive payment or services from a third party for any aspect of the submitted work (including but not limited to grants, data monitoring board, study design, manuscript preparation, statistical analysis, etc…)?

Complete each item by typing an X in answer yes or not and completing the information requested if an answer is Yes. If you have more than one relationship, add lines.

1. Grant

___No ___ _

2. Consulting fee or honorarium

___No

3. Support for travel to meetings for the study or other purposes

___No

4. Fees for participation in review activities such as data monitoring boards, statistical analysis, end-point committees, and the like

___No

5. Payment for writing or reviewing the manuscript

___No

6. Provision of writing assistance, medicines, equipment, or administrative support

___No

7. Other

___No

* This means money that your institution received for your efforts on this study.
† Use this section to provide any needed explanation.

Section 3. Relevant financial activities outside the submitted work

1. Board membership

___French Arthroscopic Society

2. Consultancy

Yes, money paid to you
Name of entity: SBM, Move Up

3. Employment

___No

4. Expert testimony

___No

5. Grants/grants pending

___No

6. Payment for lectures including service on speakers bureaus

___Move Up

7. Payment for manuscript preparation

___No

8. Patents (planned, pending or issued)

___No

9. Royalties

___No

10. Payment for development of educational presentations

___No

11. Stock/stock options

___No __

12. Travel/accommodations/ meeting expenses unrelated to activities listed**

___No

13. Other (err on the side of full disclosure)

___No

** For example, if you report a consultancy above there is no need to report travel related to that consultancy on this line.

Section 4. Other relationships

Are there other relationships or activities that readers could perceive to have influenced, or that give the appearance of potentially influencing, what you wrote in the submitted work?

___No other relationships/conditions/circumstances that present a potential conflict of interest

>>>>>>>>>>><<<<<<<<<<<

The International Committee of Medical Journal Editors

The ICMJE Disclosure of Potential Conflicts of Interest Form was adopted by *Arthroscopy: The Journal of Arthroscopic and Related Surgery* along with 17 other leading orthopaedic journals at the 2011 annual meeting of the American Academy of Orthopaedic Surgeons.

Arthroscopy: The Journal of Arthroscopic and Related Surgery

— Instructions —

ICMJE Form for Disclosure of Potential Conflicts of Interest

Each author of the manuscript must separately complete and save this form using his or her name in the file name. Each author's completed form must then be uploaded with the manuscript.

The purpose of this form is to provide readers of your manuscript with information about your other interests that could influence how they receive and understand your work. The form is in four parts:

Section 1. Identifying information

Enter your full name and provide the manuscript title.

Section 2. The work under consideration for publication

This section asks for information about the work that you have submitted for publication. The time frame for this reporting is that of the work itself, from the initial conception and planning to the present. The requested information is about resources that you received, either directly or indirectly (via your institution), to enable you to complete the work. Checking "No" means that you did the work without receiving any financial support from any third party — that is, the work was supported by funds from the

same institution that pays your salary and that institution did not receive third-party funds with which to pay you. If you or your institution received funds from a third party to support the work, such as a government granting agency, charitable foundation, or commercial sponsor, check "Yes." Then complete the provide the information requested.

Section 3. Relevant financial activities outside the submitted work

This section asks about your financial relationships with entities in the biomedical arena that could be perceived to influence, or that give the appearance of potentially influencing, what you wrote in the submitted work. You should disclose interactions with ANY entity that could be considered broadly relevant to the work.

Report all sources of revenue paid (or promised to be paid) directly to you or your institution on your behalf over the 36 months prior to submission of the work. This should include all monies from sources with relevance to the submitted work, not just monies from the entity that sponsored the research. Please note that your interactions with the work's sponsor that are outside the submitted work should also be listed here. If there is any question, it is usually better to disclose a relationship than not to do so.

For grants you have received for work outside the submitted work, you should disclose support ONLY from entities that could be perceived to be affected financially by the published work, such as entities or foundations supported by entities that could be perceived to have a financial stake in the outcome. Public funding sources, such as government agencies, charitable foundations, or academic institutions, need not be disclosed. For example, if a government agency sponsored a study in which you have been involved and drugs were provided by a pharmaceutical company, you need only list the pharmaceutical company.

Section 4. Other relationships

Use this section to report other relationships or activities that readers could perceive to have influenced, or that give the appearance of potentially influencing, what you wrote in the submitted work.

ICMJE Form for Disclosure of Potential Conflicts of Interest

Section 1. Identifying Information

1. Given Name MANSAT ________
2. Surname PIERRE__________________
3. Are you the corresponding author? NO
4. Effective Date 19/08/2023___________________________
5. Manuscript Title: **Arthroscopic distal clavicle bone block combined with Hill-Sachs remplissage for primary anterior shoulder instability treatment**

Section 2. The Work Under Consideration for Publication

Did you or your institution at any time receive payment or services from a third party for any aspect of the submitted work (including but not limited to grants, data monitoring board, study design, manuscript preparation, statistical analysis, etc…)?

Complete each item by typing an X in answer yes or not and completing the information requested if an answer is Yes. If you have more than one relationship, add lines.

1. Grant

___No ___ _

2. Consulting fee or honorarium

___No

3. Support for travel to meetings for the study or other purposes

___No

4. Fees for participation in review activities such as data monitoring boards, statistical analysis, end-point committees, and the like

___No

5. Payment for writing or reviewing the manuscript

___No

6. Provision of writing assistance, medicines, equipment, or administrative support

___No

7. Other

___No

* This means money that your institution received for your efforts on this study.
† Use this section to provide any needed explanation.

Section 3. Relevant financial activities outside the submitted work

1. Board membership

___No

2. Consultancy

No

3. Employment

___No

4. Expert testimony

___No

5. Grants/grants pending

___No

6. Payment for lectures including service on speakers bureaus

___No

7. Payment for manuscript preparation

___No

8. Patents (planned, pending or issued)

___No

9. Royalties

___No

10. Payment for development of educational presentations

___No

11. Stock/stock options

___No __

12. Travel/accommodations/ meeting expenses unrelated to activities listed**

___No

13. Other (err on the side of full disclosure)

___No

** For example, if you report a consultancy above there is no need to report travel related to that consultancy on this line.

Section 4. Other relationships

Are there other relationships or activities that readers could perceive to have influenced, or that give the appearance of potentially influencing, what you wrote in the submitted work?

___No other relationships/conditions/circumstances that present a potential conflict of interest

>>>>>>>>>>><<<<<<<<<<<

The International Committee of Medical Journal Editors

The ICMJE Disclosure of Potential Conflicts of Interest Form was adopted by *Arthroscopy: The Journal of Arthroscopic and Related Surgery* along with 17 other leading orthopaedic journals at the 2011 annual meeting of the American Academy of Orthopaedic Surgeons.

Arthroscopy: The Journal of Arthroscopic and Related Surgery

— Instructions —

ICMJE Form for Disclosure of Potential Conflicts of Interest

Each author of the manuscript must separately complete and save this form using his or her name in the file name. Each author's completed form must then be uploaded with the manuscript.

The purpose of this form is to provide readers of your manuscript with information about your other interests that could influence how they receive and understand your work. The form is in four parts:

Section 1. Identifying information

Enter your full name and provide the manuscript title.

Section 2. The work under consideration for publication

This section asks for information about the work that you have submitted for publication. The time frame for this reporting is that of the work itself, from the initial conception and planning to the present. The requested information is about resources that you received, either directly or indirectly (via your institution), to enable you to complete the work. Checking "No" means that you did the work without receiving any financial support from any third party — that is, the work was supported by funds from the

same institution that pays your salary and that institution did not receive third-party funds with which to pay you. If you or your institution received funds from a third party to support the work, such as a government granting agency, charitable foundation, or commercial sponsor, check "Yes." Then complete the provide the information requested.

Section 3. Relevant financial activities outside the submitted work

This section asks about your financial relationships with entities in the biomedical arena that could be perceived to influence, or that give the appearance of potentially influencing, what you wrote in the submitted work. You should disclose interactions with ANY entity that could be considered broadly relevant to the work.

Report all sources of revenue paid (or promised to be paid) directly to you or your institution on your behalf over the 36 months prior to submission of the work. This should include all monies from sources with relevance to the submitted work, not just monies from the entity that sponsored the research. Please note that your interactions with the work's sponsor that are outside the submitted work should also be listed here. If there is any question, it is usually better to disclose a relationship than not to do so.

For grants you have received for work outside the submitted work, you should disclose support ONLY from entities that could be perceived to be affected financially by the published work, such as entities or foundations supported by entities that could be perceived to have a financial stake in the outcome. Public funding sources, such as government agencies, charitable foundations, or academic institutions, need not be disclosed. For example, if a government agency sponsored a study in which you have been involved and drugs were provided by a pharmaceutical company, you need only list the pharmaceutical company.

Section 4. Other relationships

Use this section to report other relationships or activities that readers could perceive to have influenced, or that give the appearance of potentially influencing, what you wrote in the submitted work.

ICMJE Form for Disclosure of Potential Conflicts of Interest

Section 1. Identifying Information

1. Given Name MARTINEL ________
2. Surname VINCENT__________________
3. Are you the corresponding author? NO
4. Effective Date 19/08/2023____________________________
5. Manuscript Title: **Arthroscopic distal clavicle bone block combined with Hill-Sachs remplissage for primary anterior shoulder instability treatment**

Section 2. The Work Under Consideration for Publication

Did you or your institution at any time receive payment or services from a third party for any aspect of the submitted work (including but not limited to grants, data monitoring board, study design, manuscript preparation, statistical analysis, etc…)?

Complete each item by typing an X in answer yes or not and completing the information requested if an answer is Yes. If you have more than one relationship, add lines.

1. Grant

___No ___ _

2. Consulting fee or honorarium

___No

3. Support for travel to meetings for the study or other purposes

___No

4. Fees for participation in review activities such as data monitoring boards, statistical analysis, end-point committees, and the like

___No

5. Payment for writing or reviewing the manuscript

___No

6. Provision of writing assistance, medicines, equipment, or administrative support

___No

7. Other

___No

* This means money that your institution received for your efforts on this study.
† Use this section to provide any needed explanation.

Section 3. Relevant financial activities outside the submitted work

1. Board membership

___French Society of Shoulder and Elbow

2. Consultancy

Yes, money paid to you
Name of entity: SBM, Move Up

3. Employment

___No

4. Expert testimony

___No

5. Grants/grants pending

___No

6. Payment for lectures including service on speakers bureaus

___No

7. Payment for manuscript preparation

___No

8. Patents (planned, pending or issued)

___No

9. Royalties

___No

10. Payment for development of educational presentations

___No

11. Stock/stock options

___No __

12. Travel/accommodations/ meeting expenses unrelated to activities listed**

___No

13. Other (err on the side of full disclosure)

___No

** For example, if you report a consultancy above there is no need to report travel related to that consultancy on this line.

Section 4. Other relationships

Are there other relationships or activities that readers could perceive to have influenced, or that give the appearance of potentially influencing, what you wrote in the submitted work?

___No other relationships/conditions/circumstances that present a potential conflict of interest

>>>>>>>>>>><<<<<<<<<<<

The International Committee of Medical Journal Editors

The ICMJE Disclosure of Potential Conflicts of Interest Form was adopted by *Arthroscopy: The Journal of Arthroscopic and Related Surgery* along with 17 other leading orthopaedic journals at the 2011 annual meeting of the American Academy of Orthopaedic Surgeons.

Arthroscopy: The Journal of Arthroscopic and Related Surgery

— Instructions —

ICMJE Form for Disclosure of Potential Conflicts of Interest

Each author of the manuscript must separately complete and save this form using his or her name in the file name. Each author's completed form must then be uploaded with the manuscript.

The purpose of this form is to provide readers of your manuscript with information about your other interests that could influence how they receive and understand your work. The form is in four parts:

Section 1. Identifying information

Enter your full name and provide the manuscript title.

Section 2. The work under consideration for publication

This section asks for information about the work that you have submitted for publication. The time frame for this reporting is that of the work itself, from the initial conception and planning to the present. The requested information is about resources that you received, either directly or indirectly (via your institution), to enable you to complete the work. Checking "No" means that you did the work without receiving any financial support from any third party — that is, the work was supported by funds from the

same institution that pays your salary and that institution did not receive third-party funds with which to pay you. If you or your institution received funds from a third party to support the work, such as a government granting agency, charitable foundation, or commercial sponsor, check "Yes." Then complete the provide the information requested.

Section 3. Relevant financial activities outside the submitted work

This section asks about your financial relationships with entities in the biomedical arena that could be perceived to influence, or that give the appearance of potentially influencing, what you wrote in the submitted work. You should disclose interactions with ANY entity that could be considered broadly relevant to the work.

Report all sources of revenue paid (or promised to be paid) directly to you or your institution on your behalf over the 36 months prior to submission of the work. This should include all monies from sources with relevance to the submitted work, not just monies from the entity that sponsored the research. Please note that your interactions with the work's sponsor that are outside the submitted work should also be listed here. If there is any question, it is usually better to disclose a relationship than not to do so.

For grants you have received for work outside the submitted work, you should disclose support ONLY from entities that could be perceived to be affected financially by the published work, such as entities or foundations supported by entities that could be perceived to have a financial stake in the outcome. Public funding sources, such as government agencies, charitable foundations, or academic institutions, need not be disclosed. For example, if a government agency sponsored a study in which you have been involved and drugs were provided by a pharmaceutical company, you need only list the pharmaceutical company.

Section 4. Other relationships

Use this section to report other relationships or activities that readers could perceive to have influenced, or that give the appearance of potentially influencing, what you wrote in the submitted work.

ICMJE Form for Disclosure of Potential Conflicts of Interest

Section 1. Identifying Information

1. Given Name BALTASSAT ________
2. Surname ANTOINE__________________
3. Are you the corresponding author? NO
4. Effective Date 19/08/2023___________________________
5. Manuscript Title: **Arthroscopic distal clavicle bone block combined with Hill-Sachs remplissage for primary anterior shoulder instability treatment**

Section 2. The Work Under Consideration for Publication

Did you or your institution at any time receive payment or services from a third party for any aspect of the submitted work (including but not limited to grants, data monitoring board, study design, manuscript preparation, statistical analysis, etc…)?

Complete each item by typing an X in answer yes or not and completing the information requested if an answer is Yes. If you have more than one relationship, add lines.

1. Grant

___No ___ _

2. Consulting fee or honorarium

___No

3. Support for travel to meetings for the study or other purposes

___No

4. Fees for participation in review activities such as data monitoring boards, statistical analysis, end-point committees, and the like

___No

5. Payment for writing or reviewing the manuscript

___No

6. Provision of writing assistance, medicines, equipment, or administrative support

___No

7. Other

___No

* This means money that your institution received for your efforts on this study.
† Use this section to provide any needed explanation.

Section 3. Relevant financial activities outside the submitted work

1. Board membership

___No

2. Consultancy

No

3. Employment

___No

4. Expert testimony

___No

5. Grants/grants pending

___No

6. Payment for lectures including service on speakers bureaus

___No

7. Payment for manuscript preparation

___No

8. Patents (planned, pending or issued)

___No

9. Royalties

___No

10. Payment for development of educational presentations

___No

11. Stock/stock options

___No __

12. Travel/accommodations/ meeting expenses unrelated to activities listed**

___No

13. Other (err on the side of full disclosure)

___No

** For example, if you report a consultancy above there is no need to report travel related to that consultancy on this line.

Section 4. Other relationships

Are there other relationships or activities that readers could perceive to have influenced, or that give the appearance of potentially influencing, what you wrote in the submitted work?

___No other relationships/conditions/circumstances that present a potential conflict of interest

>>>>>>>>>>><<<<<<<<<<<

The International Committee of Medical Journal Editors

The ICMJE Disclosure of Potential Conflicts of Interest Form was adopted by *Arthroscopy: The Journal of Arthroscopic and Related Surgery* along with 17 other leading orthopaedic journals at the 2011 annual meeting of the American Academy of Orthopaedic Surgeons.
